# Lessons from SARS-CoV, MERS-CoV, and SARS-CoV-2 Infections: What We Know So Far

**DOI:** 10.1155/2022/1156273

**Published:** 2022-08-12

**Authors:** Radi Taha Alsafi

**Affiliations:** Department of Laboratory Medicine, Faculty of Applied Medical Sciences, Umm Al-Qura University, Makkah, Saudi Arabia

## Abstract

Within past decades, human infections with emerging and reemerging zoonotic viral pathogens have raised the eminent public health concern. Since November 2002, three highly pathogenic and major deadly human coronaviruses of the *βετα*-genera (*β*-hCoVs), namely, severe acute respiratory distress syndrome-coronavirus (SARS-CoV), middle east respiratory syndrome-coronavirus (MERS-CoV), and SARS-CoV-2, have been globally emerged and culminated in the occurrence of SARS epidemic, MERS outbreak, and coronavirus disease 19 (COVID-19) pandemic, respectively. The global emergence and spread of these three major deadly *β*-hCoVs have extremely dreadful impacts on human health and become an economic burden. Unfortunately, clear specific and highly efficient medical countermeasures for these three *β*-hCoVs and their underlying fatal illnesses remain under development. Although they belong to the same family and share many features and convergent evolution, these three deadly *β*-hCoVs have some important and obvious differences. By utilizing their lessons and gaining a deeper understanding of these *β*-hCoVs, we can identify areas of improvement and provide preparedness plans for fighting and controlling the future reemerging human infections that might arise from them or from other potential pathogenic hCoVs. Therefore, this review summarizes the state-of-the-art information and compares the similarities and dissimilarities between SARS-CoV, MERS-CoV, and SARS-CoV-2, in terms of their evolution trait, genome organization, host cell entry mechanisms, tissue infectivity tropisms, transmission routes and contagiousness, and the clinical characteristics, laboratory features, and immunological abnormalities of their related illnesses. It also provides an overview of the emerging SARS-CoV-2 variants. Additionally, it discusses the challenges of the most proposed treatment options for SARS-CoV-2 infections.

## 1. Introduction

Coronaviruses (CoVs) refer to a highly diverse group of enveloped, nonsegmented, positive-sense, and single-stranded RNA viruses with crown-like thorns on their surface. They belong to the *Coronavirinae* subfamily in the *Coronaviridae* family of the *Nidovirales* order [1]. According to their genomic characteristics and branching in the phylogenetic tree, CoVs have been divided into four genera: *Alphacoronaviruses* (*α*-CoVs), *Betacoronaviruses* (*β*-CoVs), *Gammacoronaviruses* (*γ*-CoVs), and *Deltacoronaviruses* (*δ*-CoVs) [2]. Of these four genera, only members of the *α*- and *β*-genera CoVs attract more attention because of their ability to infect humans and different animal species and cross the animal–human barriers [1, 2]. As yet, seven documented types of CoVs are familiar to infect humans (hCoVs) and include two *α*-genera (HCoV-NL63 and HCoV-229E) and five *β*-genera [HCoV-OC43, HCoV-HKU1, severe acute respiratory syndrome coronavirus (SARS-CoV), middle east respiratory syndrome coronavirus (MERS-CoV), and the latest emerged severe acute respiratory syndrome coronavirus-2 (SARS-CoV-2)] [3, 4]. Of these, hCoVs, HCoV-NL63, HCoV-229E, HCoV-OC43, and HCoV-HKU1 are recognized to mainly cause asymptomatic or mild respiratory and gastrointestinal symptoms, accounting for 5%–30% of common colds among humans [3, 4].

Table 1 shows that, between 2002 and 2012, two highly pathogenic hCoVs of *β*-genera (*β*-hCoVs), namely, SARS-CoV and MERS-CoV, emerged and caused dreadful threats to human health [5]. SARS-CoV infection firstly emerged in Guangdong province, China, and from where it spread globally to result in an outbreak nominated as severe acute respiratory syndrome (SARS) epidemic. From November 2002 to August 2003, over 8,000 individuals in 32 countries around the world had been infected with SARS, of which 20%–30% necessitated hospitalization and 9.6% died [6, 7]. Chronologically, no more SARS-infected cases have been reported since May 2004 [2, 8]. Ten years later, the second outbreak of *β*-hCoVs, termed the Middle East Respiratory Syndrome (MERS) outbreak and caused by MERS-CoV infection, emerged in June 2012 in Jeddah, Saudi Arabia [9], and then transmitted to Asia and other countries around the world [10, 11]. MERS-CoV spread over 27 states. It was less panic and infected fewer people than SARS-CoV. However, it had a higher case fatality rate of 34.3% [11–13]. Nonetheless, a laboratory-confirmed case of MERS infection was recently reported, indicating its persistent endemicity in causing sporadic respiratory in some countries within and outside the Middle East regions [13]. From its first emergence in 2012 to December 2019, 2,499 laboratory-confirmed cases of MERS-CoV infection, including 858 deaths, were reported from 27 countries, of which 2,106 cases and 780 deaths were from Saudi Arabia [13]. Coherently, the World Health Organization (WHO) had placed all members of *β*-hCoVs on the priority of human viral pathogens [12]. Surprisingly, this prescience of WHO has been latterly proved on 31 December 2019, whereas the third infection with a novel member of *β*-hCoVs was named severe acute respiratory syndrome coronavirus 2 (SARS-CoV-2). The first case of SARS-CoV-2 infection was reported in the city of Wuhan, Hubei province, China [14], and it has since disseminated swiftly and aggressively all over the world in a short period, resulting in its documented global pandemic (i.e., COVID-19 pandemic) as declared on 11 March 2020 by WHO, making it the first hCoV to cause a pandemic [15]. Even though SARS-CoV-2 had a lower case fatality rate than SARS-CoV and MERS-CoV, it had much higher transmissibility and contagiousness rates [16] and had a higher catastrophic effect on the whole world, considering its emergence as the most sequential global health crisis since the epoch of the influenza pandemic of 1918 [16, 17].

The clinical picture of SARS, MERS, and COVID-19 varies widely from mild respiratory symptoms to fatal respiratory and extra-respiratory complications, depending upon infection severity and the patient's immune status [12–17]. To this end, patients with severe forms of SARS, MERS, or COVID-19 disease usually develop vigorous immunological and systemic hyperinflammatory abnormalities, leading to a rapid clinical deterioration of their health status with the development of fatal acute lung injury (ALI), acute respiratory distress syndrome (ARDS), and cardiovascular and other multiorgan damage [18, 19]. As of 25 April 2022, the confirmed global infections of COVID-19 are over 505 million human cases, including more than 6.2 million confirmed deaths (WHO COVID-19 dashboard (https://www.gavi.org).

Although SARS-CoV, MERS-CoV, and SARS-CoV-2 belong to the same family of hCoVs and share convergent evolution and many features and their related global outbreaks and pandemic pose dreadful threats to human health, they differ from one another. Thus, this review aims to recapitulate the cutting-edge knowledge and provide an update on the major similar and diverse features of these three hCoVs and their related infections and lethal illnesses. It further provides an overview of the reported new variants of SARS-CoV-2 and the challenges of the proposed therapeutic approaches for the treatment of COVID-19 patients.

## 2. Major Similar and Dissimilar Features of SARS-CoV, MERS-CoV, and SARS-CoV-2 Infections

### 2.1. Theories of Evolution Trait

Histories of the first emerging cases of pathogenic CoVs were remarkably related to interactions between humans and animal hosts. In this context, bats and rodents have been proposed as the primary reservoir sources of pathogenic human *β*-CoVs and *α*-CoVs, whereas avian species are the main sources of *γ*-CoVs and *δ*-CoVs [20]. Backing the hypothesis that potential pathogenic CoVs could cross the species barrier, SARS-CoV and MERS-CoV are thought to originate in bats as intermediate hosts and be transmitted to humans from infected market civets and subsequently human-to-human transmission [21, 22]. In support, a strain of CoV shared 99.8% homological similarity with human SARS-CoV at the nucleotide sequence level was successfully isolated from palm civets from wild animal markets in southern China in October 2003, believing that palm civets were important intermediate hosts for human SARS-CoV [23]. Likewise, a MERS-CoV strain was isolated from bat stool and shared 100% RNA nucleotide identity with that of MERS-CoV isolated from a MERS patient [13]. Moreover, a bat-CoV with close phylogenetic similarity with human-MERS-CoV and the ability of bats were also demonstrated [24], indicating bats were the major probable reservoirs for human-MERS-CoV. The intermediate reservoir roles of dromedary camels in SARS-CoV and MERS-CoV transmission to humans have also been evidenced [25]. For instance, MERS-CoV was successfully isolated from the dromedary camels in Saudi Arabia [26] and Qatar [27], showing 99.2%–99.8% genetic identity sharing with the human MERS-CoV. Compared to SARS-CoV and MERS-CoV, the origin of SARS-CoV-2 seems more sophisticated. SARS-CoV-2 probably also emerged from bats to intermediate hosts such as minks and pangolins and then was transmitted to humans [28]. Supposing a hypothesis that bats and pangolins might be the primary natural reservoirs for the emerging SARS-CoV-2, virological studies have successfully detected a great genetic similarity between the human SARS-CoV-2 emerging in Wuhan, China, and a CoV isolated from bats (Bat-CoV RaTG13) [29] and pangolins (pangolin-CoV) [30]. Other animal hosts have also been speculated to be the probable origin and intermediate reservoirs for SARS-CoV-2 [31, 32].

### 2.2. Genome Organization

Genomic knowledge of the clinically significant hCoVs firmly promotes a better understanding of their origin, pathogenesis, and virulence. In this era, each SARS-CoV and MERS-CoV has around 29.75  and 30.11 kb genomic size, respectively, whereas the genome size of SARS-CoV-2 is around 29.9 kb, indicating that MERS-CoV has the largest genomic size, followed by SARS-CoV-2 and SARS-CoV [33, 34]. However, these three viruses have a typical genomic structure composed of 5′ methylated cap-leader-untranslated region (UTR); a genetic region encoding 16 non-structural and 5–8 accessory proteins; a genetic region encoding the four main structural proteins [spike (S), envelope (E), membrane (M), and nucleocapsid (N)], which are collectively critical for viral life cycle, and 3′ UTR-poly (A) tail scheme [35, 36]. The primary function of the S protein, which is subcleaved into S1 and S2 fractions, is to bind to the targeted viral receptors on the host's cell surfaces receptor through the receptor-binding domain (RBD) of its S1 fraction, whereas the S2 fraction comprises a fusion protein (FP) that mediates membrane fusion and penetration of the whole viral genome into the cytoplasm of the host cells [37]. The N protein functionally has several activities in mediating intracellular viral replication processes, whereas the M and E proteins are critically involved in the assembly and release processes of the newly formed viral particles [38]. The genomic comparative studies have shown that SARS-CoV-2 has approximately 79.5% and 50% genomic homology with SARS-CoV and MERS-CoV, respectively [35, 39]. Additionally, there is approximately 76%–78% sequence homology between the overall amino acids of the S protein for SARS-CoV-2 and SARS-CoV. However, the genetic materials encoding the S protein of SARS-CoV-2 have a higher mutation willingness than those of SARS-CoV and MERS-CoV [40, 41]. Additionally, there is now much interest in identifying specific molecular characteristics to reclassify and differentiate the different genera/lineages of hCoVs. With this concept, the members of *β*-hCoVs have been subclassified into four subgenera, namely, *Sarbecoviruses*, *Merbecoviruses*, *Nobecoviruses*, and *Embecoviruses*, of which SARS-CoV and SARS-CoV-2 are following *Sarbecoviruses* subgenera, whereas MERS-CoV is a member of the *Merbecoviruses* subgenera [42].

### 2.3. Transmission Routes and Contagiousness

In terms of contagiousness, SARS-CoV-2 has the highest transmissibility behavior, followed by SARS-CoV and MERS-CoV [43], and there are various transmission routes for human infections with these potential pathogenic hCoVs [32, 36, 43]. On this point, SARS-CoV is primarily transmitted by inhaling infected air droplets through close human-to-human contact and contact with contaminated surfaces and healthcare devices [44, 45]. A contaminated fecal-oral transmission route was also supposed [46]. Person-to-person transmission is also the key source of MERS-CoV infection [47]. Additionally, MERS-CoV has been isolated from serum, cerebrospinal fluid, stool, vomitus, and urine specimens of MERS patients [13]. Likewise, close human-to-human contact, inhalation of infected droplets, and direct contact with contaminated surfaces have been concluded as the major transmission paths of SARS-CoV-2 infection [48, 49]. Importantly, research has reported the maternal-fetal vertical transmission path of SARS-CoV-2 infection [50], and as evidence of gastrointestinal infection, live SARS-CoV-2 and its nucleocapsid protein were isolated from the stool specimens and intestinal tissues [51, 52]. SARS-CoV-2 was also isolated from blood, sputum, saliva, urine, ocular fluids, and aerosol specimens from COVID-19 patients [51–53].

### 2.4. Host Cell Entry Mechanisms and Cellular Infectivity Tropisms

The cell entry of all clinically significant pathogenic hCoVs, including SARS-CoV, MERS-CoV, and SARS-CoV-2, is primarily mediated by binding these viruses with specific functional receptors on the host's cell surfaces. The cellular distribution and expression density of these functional receptors is critically implicated in the virulence, tissue tropism, and the whole pathogenicity of their binding viruses [54, 55]. In this context, the angiotensin-converting enzyme 2 (ACE2) has been recognized as the primary host cell surface receptor for SARS-CoV [56] and SARS-CoV-2 [54, 55], whereas the dipeptidyl peptidase 4 (DPP4), also termed CD26, is the primary host cell surface receptors for MERS-CoV [57]. There is no structural sharing or sequence homology between ACE2 and DPP4 receptors [58]. The binding affinity of SARS-CoV-2 to ACE2 receptors is estimated as 10–20 times higher than that of SARS-CoV [59, 60], and this variation is attributed to differences in the (RBD) of the viruses S proteins [41, 61]. After receptor binding, the intracellular entry of the whole genome of SARS-CoV, MERS-CoV, and SARS-CoV-2 is facilitated and accomplished by priming of the viral S2 protein by the host cell transmembrane serine protease type 2 (TMPRSS2) and endosomal cysteine proteases cathepsin B/L [3, 54, 55, 59]. Thus, specific TMPRSS2 inhibitors have been proposed as a possible promising therapeutic strategy against these potential pathogenic hCoVs [3, 54, 61].

In terms of tissue infectivity tropism, it has proved that the ACE2 receptors have a vast tissue bio-distribution and are abundantly expressed in the airway ciliated epithelial cells, alveolar type II cells, epithelial cells of nasal cavity and oral mucosa, olfactory neuroepithelium, upper GIT epithelial cells, and the endothelial cells of blood vessels, heart, and small intestine [62, 63]. The tissue bio-distribution density of these ACE2 receptors is consistent with disease progression and severity in both SARS and COVID-19 infected patients [62–64]. The DPP4 receptors, however, are mainly expressed in cells of the lower respiratory airway, the kidneys, and GIT, and this may likely explain why patients with MERS have a prominent renal injury and GIT manifestations besides the clinical features of their severe atypical pneumonia [58]. Besides, DPP4 have also been found to be expressed in the thymus, liver, prostate, and bone marrow [13]. In addition to the critical role of ACE2 receptors, and as evidence for its multimodal mechanisms to invade human cells, SARS-CoV-2 can also bind to cellular neuropilin-1 (NRP1) receptors [65], integrins [66], and CD147 spike structure [67], as well as to the *β*-chains of human erythrocyte porphyrins [68]. Likewise, the transmembrane CD209L (L-SIGN) and CD209 (DC-SIGN) cellular structures have been prosed as functional co- or alternative receptors for SARS-CoV to invade human cells [69, 70]. Taking all together, there are multiple modes for these potentially pathogenic hCoVs to invade multiple types of human cells, including respiratory and extra-respiratory organs, which collectively explain why they have broad cellular and tissue infectivity tropisms than other CoVs and require more comprehensive studies to be fully elucidated.

### 2.5. Incubation Period, Clinical and Laboratory Characteristics

Knowledge of the incubation period of a potential pathogenic human virus is important in improving the surveillance, prevention, and control strategies for its disease outbreak. With this concept, the average incubation period of SARS-CoV and MERS-CoV has been estimated as 2–10  (7) days [71, 72] and 1.9–14.7 (5.5) days [72], respectively. Notably, longer incubation periods of >10 days and >20 days were, respectively, observed in a small proportion of immunocompromised SARS and MERS patients [73, 74]. In comparison, the median incubation period of SARS-CoV-2 is generally estimated as 2–14 (5.2) days 72.

At the clinical level, the mild symptomatic forms of SARS-CoV, MERS-CoV, or SARS-CoV-2 infection share a wide range of clinical manifestations, including fever, cough, malaise, sore throat, dyspnea, headache, fatigue or myalgia, and diarrhea [12, 16, 75]. Of note, during the SARS outbreak, the disease course was usually divided into two periods: an early period of (1–7 days) of respiratory manifestations, followed by a progress period (10–14 days), in which the patients' health condition more deteriorated with a case fatality rate of 9.6% [8, 12]. On the contrary, MERS usually develops severely progressed pulmonary disease [13, 26], alongside significant acute kidney injury (AKI) and renal failure incidence in more than 50% of its cases [76]. Furthermore, a high incidence of diabetes and cardiovascular medical comorbidities are also common in MERS-infected patients, explaining why MERS has a higher case fatality rate than SARS [77]. The clinical symptoms of nonsevere forms of COVID-19 are also consistent with those of SARS and MERS [78, 79]. A significant percentage of COVID-19 patients also have diarrhea, nausea, vomiting, and other gastrointestinal symptoms [80]. Consistent with SARS and MERS, the severe cases of COVID-19 have also manifested worse clinical outcomes and deaths due to rapid development of diffuse alveolar damage, ARDS, septic shock, cardiovascular and coagulopathy disorders, and fatal multiple organ failure, particularly in immune deteriorated and elderly patients [5, 12, 17, 19, 81].

Additionally, there are asymptomatic or subclinical patients who can disseminate hCoVs, posing a great challenge. For instance, SARS-CoV-2 and MERS have been detected in the clinical samples a few days before symptom onset [82–84]. Likewise, some asymptomatic COVID-19 cases have shown similar viral loads as those of symptomatic patients, implying that asymptomatic SARS-CoV-2 infected persons may be possible sources of the COVID-19 pandemic [49, 85].

The polymerase chain reaction- (PCR-) based molecular testing using viral RNA extracted from clinical samples is the standard detection method for SARS-CoV, MERS-CoV, and SARS-CoV-2 infections due to its high sensitivity, specificity, and simplicity [86]. Even though their sensitivity was generally lower than that of PCR tests, serological and antiviral antibody detection tests were predominantly used in retrospective diagnosis for SARS-CoV and MERS-CoV infections [77]. Similarly, the combination of serological detection of antibody responses to SARS-CoV-2 infections with PCR molecular testing was significant in the diagnosis and management of COVID-19 [87].

In terms of clinical laboratory findings, as demonstrated in Table 1, there are great similarities concerning the abnormal hematological and biochemical laboratory findings in SARS-CoV, MERS-CoV, and SARS-CoV-2 infected patients, including significant leukopenia, lymphopenia, thrombocytopenia, elevated serum levels of lactate dehydrogenase (LDH), and liver enzymes [aspartate aminotransferase (AST) and alanine aminotransferase (ALT)] and their diagnostic utility in determining disease severity and poorer outcomes [5, 88–91]. Cardiac and renal injuries, characterized by significant increases in serum creatine kinase (CK) and creatinine levels, respectively, are also common, particularly in MERS and COVID-19 patients [92, 93]. Consistent with this, most MERS patients have been reported to trigger profound renal failure [16, 92]. Besides, disseminated intravascular coagulopathy (DIC) associated with a significant increase in D-dimer level and prolonged time of blood coagulation tests are common among SARS, MERS, and COVID-19, especially in severely infected patients [94]. Additionally, hypoalbuminemia is frequently reported in patients with severe CoVID-19 infection [95], and hypocalcemia with undefined underlying ethology was reported in 60% of patients infected with SARS [75].

### 2.6. Host Immune Responses, Viral Immune Evasion, and Abnormal Immunological Changes

During the early phases of immune responses in patients with SARS-CoV, MERS-CoV, and SARS-CoV-2 infection, the arms of innate immunity are orchestrating to recognize these pathogens and their infected cells through the Toll-like receptors (TLRs), RIG-I-like receptors (RLRs), and other types of pathogens recognition receptors [96, 97]. Synchronously, macrophages, monocytes, neutrophils, cytotoxic CD8+ T cells, CD4+ T cells, natural killer (NK) cells, dendritic cells, and other immune cells are recruited to the site of infection to eradicate these viruses and their infected cells through multiple mechanisms [96–99]. Notably, if a defect in the host's antiviral immune response occurs due to viral and/or host variables, it subsequently delays the viral clearance and ultimately exaggerates its underlying disease [96–99]. To this end, SARS-CoV, MERS-CoV, and SARS-CoV-2 infections are usually associated with various phenotypes of lymphocytopenia and lymphocytes exhaustion and dysfunctionality, and these immunological abnormalities are closely associated with viral infection severity in their susceptible patients [5, 100, 101]. In this sense, it is particularly significant that adult patients at the early stage of COVID-19 disease often have a remarkable decrease in their CD8^+^ and CD4^+^ T-cell subsets, leukopenia, and lymphopenia, alongside an elevation in their serum levels of liver enzymes (AST and ALT) and lactic dehydrogenase (LDH) [101, 102]. Furthermore, the neutrophil-to-lymphocyte ratio (NLR) has been identified as a predictive factor for early-stage prediction and critical illness in COVID-19-infected patients [103].

Besides their functional exhaustion effects on antiviral lymphocytes in their infected patients, each of SARS-CoV, MERS-CoV, and SARS-CoV-2 has been evolutionarily acquiring an ability to encode numerous proteins to impair type-1 interferon-mediating antiviral immunity to further evade from the host immune defense mechanisms [1, 96]. For instance, the open reading frame (ORF) and N protein of SARS-CoV directly suppress IFN-1 antiviral signaling [104], and MERS-CoV can shift antiviral IFN-1 and nuclear factor-*κ*B signaling pathways to proinflammatory Th17 paths [105]. More recently, SARS-CoV-2 has shown marked blocking activities against antiviral IFN-1 immunity of the hosts through multiple mechanisms, including induction of defects in IFN‐I signaling activity and enhancing the formation of anti-IFN-1 autoantibodies in certain types of COVID-19 patients [106, 107].

Most importantly, several studies have disclosed the pivotal pathogenic roles of the aberrant and deviating cytokines and chemokines responses, a phenomenon known as “hypercytokinemia” or “cytokine storm,” in the development and exacerbation of fatal pulmonary and systemic hyperinflammatory syndrome in susceptible patients severely infected with SRAS-CoV, MERS-CoV, or SARS-CoV-2, including diffuse alveolar damage, ARDS, vascular damage and sepsis, coagulation and cardiac disorders, renal damage, and other extrapulmonary organs failure [108–110]. Though the underlying mechanisms are not fully understood, it is strongly thought that the extensive production of robust proinflammatory cytokines and chemokines, including interleukin-6 (IL-6), IL-1*β*, IL-7, IL-8, tumor necrosis factor-*α* (TNF-*α*), IFN-*γ*, Janus kinase (JAK) pathway, macrophage inflammatory proteins, C-reactive protein, and CXCL and CC chemokine families (e.g., CXCL1, CXCL2, CXCL8, CXCL10, CXCL17, CCL2, CCL5, and CCL20), is beyond the development of such life-threatening immunological abnormalities in severely ill patients [111–113]. The abnormally elevated serum levels and the prolonged response of the aforementioned proinflammatory cytokines and chemokines and their positive correlations with the severity and poor outcomes were markedly observed in patients infected with SARS [70, 109, 114], MERS [109, 110, 115], and COVID-19 [111, 116, 117], despite their varied immunological components and diversity. Similar to SARS-CoV [118], elevated serum levels of type 2 cytokines were also detected in SARS-CoV-2 infected patients [119]. In addition, increased lymphocyte pyroptosis and increased number of proinflammatory immune cells (e.g., CCR4+/CCR6+ Th17 cells and HLA-DR/CD38 double-positive) are also key pathogenic factors in this phenomenon [106, 113, 120]. Furthermore, SARS-CoV-2 can also induce Nlrp3*γ* inflammasome and endoplasmic reticulum-stress-mediated inflammation to trigger these life-threatening hypercytokinemia/hyperinflammatory syndromes [121].

## 3. SARS-CoV-2 New Variants and Their Possible Implications

Despite the global mass efforts to reduce the spreading and severity process of the COVID-19 pandemic, several new SARS-CoV-2 variants with multigenetic mutations have been reported worldwide [122, 123]. They have faced an increasing concern as they might pose a risk of hindering anti-SARS-CoV-2 infection vaccine effectiveness and long-term immunity [122, 123]. They are classified by the Centers for Disease Control and Prevention (CDC) and WHO in collaboration with the SARS-CoV-2 Interagency Group (SIG) into three main categories: (1) variants of concern (VOCs); (2) variants of interest (VOIs); and (3) variants of high consequence (VOHCs) [17]. Among them, VOCs, which include Alpha, Beta, Gamma, Delta, and Omicron variants, have shown multiple key mutations in the genetic materials of the viral spike protein RBD. These five VOCs have also been proposed to probably increase the virulence and disease severity of SARS-CoV-2 infections [123–127]. Moreover, they showed a remarkable reduction in neutralization by monoclonal antibodies, convalescent plasma, and postvaccination sera treatments; thus, their reemergent infection may further threaten SARS-CoV-2 infections [128, 129]. The nomenclatures and the different characters of all reported SARS-CoV-2 new variants are summarized in Table 2.

## 4. Proposed Therapeutic Options for COVID-19 Disease and Their Challenges

**Figure 1:**
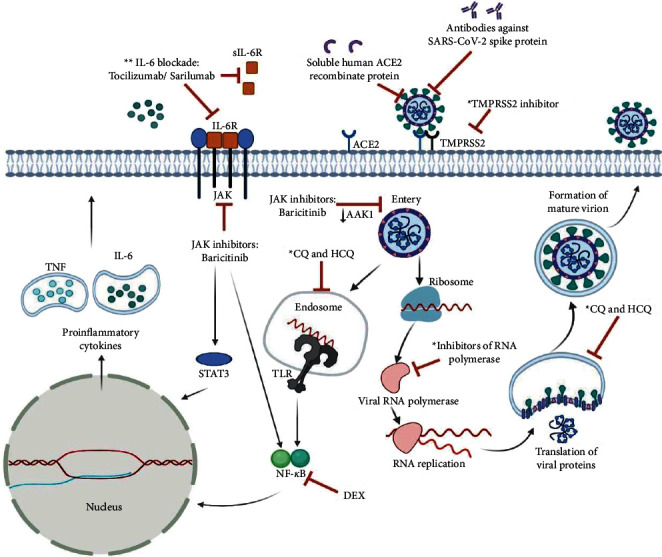
An illustration of the proposed therapeutic targets for the treatment of SARS-COV-2 infections. Angiotensin-converting enzyme-2 (ACE2); AP2-associated protein kinase 1 (AAK1); chloroquine (CQ); dexamethasone (DEX); hydroxychloroquine (HCQ); interleukin (IL-); Janus kinase (JAK); nuclear factor-*κ*B (NF-⎢B); signal transducer and activator of transcription 3 (STAT3); soluble IL-6 receptor (sIL-6R); transmembrane serine protease-2 (TMPRSS2); toll-like receptor (TLR); tumor necrosis factor (TNF). Some of the potential therapeutic targets of COVID-19 have been tested *in vitro* (^*∗*^) and *in vivo* (^*∗∗*^). The figure is created with BioRender (https://biorender.com).

Since its emergence, a variety of therapeutic options (Figure 1) have been proposed for the management and control of the COVID-19 crisis. However, no drugs are validated yet to have significant efficiency and distinctive safety/efficacy profile in large-scale trials [17, 130]. Generally, the prosed therapeutic trials are primarily based on using direct-acting antiviral drugs (DAADs) in combination with immunomodulatory/anti-inflammatory agents, monoclonal antibody therapy, convalescent plasma therapy, and anticoagulant therapy [130]. In this concept, DAADs, such as remdesivir, molnupiravir, and favipiravir, work as direct inhibitors for viral RNA-dependent RNA polymerase (RdRp) and impede SARS-CoV-2 replication *in vitro* and *in vivo*, and have been assumed as the most potential and hopeful agents in the treatment of COVID-19 [131]. Remdesivir (GS-5734), a broad-spectrum antiviral ATP nucleotide analog, is the first RdRp inhibitor approved by the Food and Drug Administration (FDA) on 1 May 2020 for treating COVID-19 in adults and children of more than 12 years of age [132]. The European Medicines Agency has also recommended remdesivir in the emergency treatment of severely ill COVID-19 patients [133]. Molnupiravir (EIDD-2801) was authorized by The United Kingdom as an orally administered antiviral RdRp inhibitor for the treatment of adult patients with mild-to-moderate forms of COVID-19 [134]. The FDA has also issued an antiviral protease inhibitor, paxlovid, as an oral combination pill of two antiviral agents (ritonavir/nirmatrelvir), for the treatment of patients with mild-to-moderate COVID-19 [131]. In terms of clinical benefits and outcomes, all these tested DAADs have demonstrated benefits in shortness of patients' hospitalization and mechanical ventilation dependency. However, their beneficial effects against COVID-19 severity and associated mortalities remain uncertain [130, 131]. Of note, the clinical utility of these direct-acting and antibody-based antiviral treatments is more effective during the early phase of the clinical course of the COVID-19 illness when SARS-CoV-2 replication is greatest [17]. Contradiction results have also been reported in some clinical trials and attributed to genetic reasons and differences in the study designs and sample sizes [135]. Furthermore, several adverse effects (e.g., nausea, vomiting, hepatic toxicity, and rectal hemorrhage) of remdesivir and other tested DAAD have been widely reported [136]. Antimalarial drugs, such as chloroquine (CQ) and hydroxychloroquine (HCQ), have also been tested to interfere with the steps of endosome-mediated viral entry and late stages of replication of SARS-CoV-2. However, their overall clinical benefits for the treatment of hospitalized COVID-19 patients remain doubtful due to a lack of efficacy and increased incidence of cardiac adverse events [17]. With respect to the value of therapeutic combination strategies, a significant number of clinical trials have revealed the favorable augmenting and superior anti-COVID-19 therapeutic effects of combining anti-SARS-CoV-2 DAADs (e.g., remdesivir) with one or more of the following direct blockers for SARS-CoV-2 cell entry and infectivity: (a) baricitinib, as a Janus kinase inhibitor, for hospitalized adults patients [137, 138]; (b) REGN-COV2 (casirivimab-imdevimab), as a specific monoclonal antibody cocktail against SARS-CoV-2 spike protein [139]; (c) epicatechin, as an inhibitor for SARS-CoV-2/ACE2 binding [140]; or (d) alpha-1 antitrypsin, as a specific inhibitor for SARS-CoV-2/TMPRSS2 interaction [141]. For instance, there is solid evidence that Janus Kinase (JAK1 and JAK2) signaling pathway is crucially involved in the induction and exacerbation of hyperinflammatory syndromes in COVID-19 patients and, in turn, specific JAK inhibitors (e.g., baricitinib and rituxolitinib) may have a particular value in repressing COVID-19 severity [142, 143]. Additionally, alveolar epithelial cells are prone to SARS-CoV-2 endocytosis mediated by the protein kinase 1 (AAK1) JAK pathway. Its block by JAK inhibitors may add further preventing activity against the cellular infectivity of SARS-CoV-2 [142, 143]. Furthermore, in order to overcome the fatal phenomenon of cytokine storm and systemic hyperinflammation in the COVID-19 crisis, the add-on therapy with an appropriate immunomodulatory-anti-inflammatory agent has been strongly suggested. Toward this aim, corticosteroids (e.g., dexamethasone), as powerful anti-inflammatory and immunosuppressive agents, have shown benefits in calming cytokine storm and inflammation-mediated lung injury in some patients with severe COVID-19 [137, 144]. Nevertheless, the nonspecific immunosuppressive effects of corticosteroids may hinder SARS-CoV-2 clearance, obstruct host antiviral immunity, and increase infectivity with other respiratory pathogens [145]. Alternatively, the advent of more specific cytokines-targeting immunomodulatory agents for silencing hypercytokinemia and its associated multi-inflammatory organ injuries and without hindering antiviral host immune protection has become an essential demand. For instance, as IL-6 is the key driver of the inflammatory state associated with COVID-19, therapeutic approaches targeting IL-6 have attracted high levels of interest, for example, antibodies that specifically block IL-6 receptors and are approved by FDA (e.g., tocilizumab and sarilumab) are under clinical trials [146]. In parallel, TNF is present in excess amounts in blood and diseased tissues of COVID-19 patients. Therefore, trials of anti-TNF-based therapy (e.g., using adalimumab, etanercept, or golimumab monoclonal antibody) have also been suggested for hospitalized COVID-19 patients [147].

## 5. Authorized COVID-19 Vaccines

In addition to the abovementioned treatment options, many efforts have been made worldwide to develop an efficient vaccine against the original SARS-CoV-2 to control the COVID-19 pandemic and repress its severity, hospitalization, and deaths. According to WHO, more than 200 vaccine candidates have been developed worldwide with various levels of efficiencies and protection duration [148, 149]. To date, the authorized and clinically implemented COVID-19 vaccines could be categorized into (i) mRNA vaccines, such as mRNA-1273 (Moderna) and BNT162b2 (Pfizer-BioNTech); (ii) nonreplicating viral vectors-based vaccines, such as AZD1222ChAdOx1 nCoV-19 vaccine (AstraZeneca), Ad26.COV2.S (JNJ-78436735, Johnson & Johnson), Ad5-nCoV (Convidicea), and Sputnik V; (iii) inactivated virus-based vaccines, such as CoronaVac (Sinovac), BBIBP-CorV (Sinopharm), Covaxin in India; KoviVac in Russia, and COVIran Barakat in Iran; and (iv) protein subunits-based vaccines, such as EpiVacCorona and ZF2001 [149–151]. Notably, the effectiveness and duration of protections of these authorized COVID-19 vaccines vary depending on the vaccine type, its manufacturer and composition, and its dosage schedule [151].

## 6. Conclusion and Future Challenges and Prospects

To date, a total of three outbreaks of lethal human infections caused by three emerged members of potential pathogenic hCoVs of the *β*–genera (SARS-CoV, MERS-CoV, and SARS-CoV-2) have raised great public health concern globally. While the first two viruses did not result in a pandemic, the third and the most recent one has culminated in a pandemic, officially named the “COVID-19 pandemic” and considered the most sequential global health crisis since the epoch of the influenza pandemic of 1918. Despite originating from the same genus and sharing many features, the three viruses have presented various important dissimilarities from one another. Compared to SARS-CoV and MERS-CoV, SARS-CoV-2 has a much higher transmissibility and contagiousness behavior. It can invade human cells through multiple mechanisms and is more prone to develop rapid genetic mutations. In order to identify areas for improvement to fight and manage their possible future reemergent outbreaks, it is critical to gain a deeper understanding of their different characteristic, pathobiological, and clinical features. At a constant line, the underlying phenotypes of the immunological abnormalities and the consequent hyperinflammatory responses are highly complex and varied among SARS, MERS, and COVID-19 patients. Thus, the establishment of more specific clinical diagnostic tools targeting the immunological changes and their downstream inflammation avenues in routine laboratories, as well as the periodic genomic analysis and close monitoring of hCoVs samples from positively infected patients to detect and characterize any newly emerging variants, are of fundamental emphases in controlling hCoVs outbreaks. In conjunction, as there are no specific medical countermeasures for these hCoVs, the establishment of more comprehensive preclinical studies in nonhuman primates and other experimental animals adapted for viruses are critical medical demand to improve the therapeutic efficacy and safety profiles of specific medical countermeasures for the diseases caused by these lethal hCoVs and improve the efficacy and safety of their overall vaccinology.

## Figures and Tables

**Table 1:** A comparative overview of SARS-CoV, MERS-CoV, and SARS-CoV-2 [2, 5, 7, 12, 13, 16, 41, 45, 49, 71–74].

Parameter	SARS-CoV	MERS-CoV	SARS-CoV2
First emergence (date)	16 November 2002	4 April 2012	7 December 2019
Virus identification (date)	March 2003	June 2012	January 2020
Causative agent declaration (date)	April 2003	September 2012	January 2020
Viral nucleotides length (kb)	29.75	30.11	29.9
Transmission triat	Animal-human human-human zoonotic viral disease	Animal-human human-human zoonotic viral disease	Animal-human human-human zoonotic viral disease
Median incubation period (days)	2–10 (7)	1.9–14.7 (5.5)	2–14 (5.2)
Induced disease (name)	SARS epidemic	MERS outbreak	COVID-19 pandemic
Confirmed global cases (N)	8096	2553	>505 million^*∗*^
Countries with confirmed infections (N)	32	27	237^*∗*^
Overall fatality rate (%)	9.6%	34.3%	2.13%
Recent status	Completely control	Sporadic continuous	Ongoing
*Frequency of associated complications*			
ARDS (%)	20%	20–30%	18–30%
AKI (%)	6.7%	41–50%	3%
*Frequency of abnormal laboratory findings in infected patients*			
Leukopenia (<4.0 × 10^9^/L) (%)	23–35%	14%	20–26.8%
Lymphopenia (<1.5 × 10^9^/L) (%)	68–85%	32%	55.3%
Thrombocytopenia (<150 × 10^9^/L) (%)	40–45%	36%	11.5–17%
High serum LDH levels (%)	50–71%	48%	43-55.5%
High serum AST levels (%)	20–30%	14%	17.9–25.3%
High serum ALT levels (%)	20–30%	11%	16.0–22.7%

ARDS: acute respiratory distress syndrome; AkI: acute kidney injury; LDH: lactate dehydrogenase; AST: aspartate aminotransferase; ALT; alanine aminotransferase. ^*∗*^According to the data released by the WHO on 25 April 2022 (WHO COVID-19 dashboard (https://www.gavi.org).

**Table 2:** SARS-CoV-2 new variants [17, 122–129].

Variant name^*∗*^	Characters and attributes
*Variants of concern (VOCs)*	
Alpha (B.1.1.7; 501Y.V1)	(i) First reported in the UK in late December 2020
	(ii) With 17 genetic mutations, including 8 in its spike protein
	(iii) With a 43–82% increase in viral transmissibility
	(iv) With an increased binding affinity to ACE2Rs

Beta (B.1.351; 501Y.V2)	(i) First reported in South Africa in October 2020
	(ii) With nine mutations in its spike protein, including three in its RBD
	(iii) With an increased binding affinity to ACE2Rs
	(iv) Escapes neutralization by MABs, convalescent, and postvaccination sera

Gamma (P.1; 501Y.V3)	(i) First reported in Brazil in December 2020
	(ii) With 10 mutations in its spike protein, including three in its RBD
	(iii) With reduced neutralization by MABs, convalescent/postvaccination sera

Delta (B.1.617.2)	(i) First reported in India in December 2020
	(ii) Caused the deadly second wave of COVID-19 in India in April 2021
	(iii) With 10 key genetic mutations in its spike protein
	(iv) Was initially considered a VOI, but due to its rapid global spreading, WHO reclassified it as a VOC in May 2021

Omicron (B.1.1.529)	(i) First reported in South Africa in November 2021
	(ii) More than 76 countries have identified Omicron variant infections
	(iii) With >30 mutations in its spike protein
	(iv) It is likely to have vaccine breakthroughs
	(v) With a 13-fold increase in viral infectivity; and its susceptibility for neutralization by MABs therapy is still an era of conflict

*Variants of interest (VOIs)*	
Epsilon (B.1.427 & B.1.429)	First emerged in the US in June 2020. It exhibits an 18.6–24% increase in transmissibility relative to wild-type preexisting strains. For this reason, the CDC to reclassify it as a VOC in the US
Zeta (P.2)	First detected in Brazil in April 2020, and it harbors eight key spike mutations
	Classified as a VOI due to its weak susceptibility for neutralization by treatments with MABs and vaccine sera
Eta (B.1.525) & Iota(B.1.526)	First emerged in the US in November 2020
	They harbor multiple spike mutations and are characterized by their potential reduction in neutralization by treatments with MABs and vaccine sera
Theta (P.3; 1092K.V1)	First detected in Japan and the Philippines in February 2021; it carries three key spike mutations
Kappa (B.1.617.1)	First detected in India in December 2021; it carries eight key spike mutations
Lambda (C.37)	First detected in Peru; due to its heightened presence in the South American region, the WHO classified it as a VOI in June 2021
Mu (B.1.62)	(i) First emerged in Columbia
	(ii) Classified as a VOI by the WHO in August 2021

*Variants of high consequences (VOHCs)*	
None	Demonstrated failure of diagnostics, significant reduction in vaccine effectiveness, and more severe clinical disease

^*∗*^The classification is as per the CDC and the WHO. ACE2Rs, angiotensin-converting enzyme 2 receptors; RBD, receptor binding domain; MABs, monoclonal antibodies.

## Data Availability

All data are included in the submitted text and tables.
